# Improved Resistive and Synaptic Characteristics in Neuromorphic Systems Achieved Using the Double-Forming Process

**DOI:** 10.3390/nano13212859

**Published:** 2023-10-28

**Authors:** Minkang Kim, Dongyeol Ju, Myounggon Kang, Sungjun Kim

**Affiliations:** 1 Division of Electronics and Electrical Engineering, Dongguk University, Seoul 04620, Republic of Koreajudongyeol0117@gmail.com (D.J.); 2 Department of Electronics Engineering, Korea National University of Transportation, Chungju-si 27469, Republic of Korea

**Keywords:** resistive switching, neuromorphic system, synapse emulation, spike-timing-dependent plasticity

## Abstract

In this study, we investigate the electrical properties of ITO/ZrO_x_/TaN RRAM devices for neuromorphic computing applications. The thickness and material composition of the device are analyzed using transmission electron microscopy. Additionally, the existence of TaON interface layers was confirmed using dispersive X-ray spectroscopy and X-ray photoelectron analysis. The forming process of the ZrO_x_-based device can be divided into two categories, namely single- and double forming, based on the initial lattice oxygen vacancies. The resistive switching behaviors of the two forming methods are compared in terms of the uniformity properties of endurance and retention. The rationale behind each I–V forming process was determined as follows: in the double-forming method case, an energy band diagram was constructed using F-N tunneling; conversely, in the single-forming method case, the ratio of oxygen vacancies was extracted based on XPS analysis to identify the conditions for filament formation. Subsequently, synaptic simulations for the applications of neuromorphic systems were conducted using a pulse scheme to achieve potentiation and depression with a deep neural network-based pattern recognition system to display the achieved recognition accuracy. Finally, high-order synaptic plasticity (spike-timing-dependent plasticity (STDP)) is emulated based on the Hebbian rule.

## 1. Introduction

Traditionally, most computers have operated based on the conventional von Neumann architecture, wherein a central processing unit (CPU) retrieves data from memory and processes information. However, in the modern era, the advancement of artificial intelligence technology has necessitated the handling of vast amounts of data, thus leading to bottleneck issues between the CPU and memory. Furthermore, the current state of complementary metal oxide semiconductor (CMOS) technology has reached its physical limits, with the performance and storage capacity of electronic devices increasingly dependent on the number of integrated transistors [1,2]. Memristors are extensively studied owing to their promising future applications as a result of their compatibility with CMOS technology, rapid response times, low-power operation, high durability, and nonvolatile memory and parallel signal processing capabilities [3]. Additionally, neuromorphic computing systems based on artificial neural networks have the potential to alleviate the aforementioned problems [4,5]. There are various types of memristors, which include magnetic random access memory [6], phase change random access memory [7], ferroelectric random access memory [8], and resistive random access memory (RRAM) [9,10,11]. The latter stands out as one of the next-generation, nonvolatile memories. RRAM, in particular, features a simple metal–insulator–metal structure with an insulator layer sandwiched between metal layers, offering the advantage of easy fabrication and rapid mass production [12]. It operates on a mechanism where conductive filaments, based on oxygen vacancies, are created and destroyed, thus resulting in the switching between the low-resistance states (LRS) and high-resistance states (HRS), respectively denoted as “On” and “Off” [13,14]. The resistance switching phenomenon in RRAM devices has been extensively explored using various binary metal oxides, such as ZrO_x_, TaO_x_, AlO_x_, TiO_x_, and HfO_x_ [15,16,17,18,19]. Among these, ZrO_x_, which has been used as the insulator layer, has gained attention as a high-K material compatible with traditional CMOS processes [20]. Previous studies reported the usage and the abilities of ZrO_x_-based resistive switching (RS) devices. Lin et al. reported a Ti/ZrO_2_/Pt resistive switching device with high-endurance cycles (>10^3^) and uniformity [21]. Additionally, Kim et al. reported that ZrO_x_ is a suitable material for use as the insulator in RRAM devices and is appropriate for applications in neuromorphic systems [22].

In this study, we aim to verify whether ZrO_x_-based memristors exhibit typical RRAM characteristics and synaptic properties for potential use in artificial synapses. Unlike most RRAM devices, the ITO/ZrO_x_/TaN device investigated herein can be formed using two methods: a single-step, single-forming (SF), and a two-step, double-forming (DF) process [23]. These two forming mechanisms are presented using energy band diagram analysis based on transmission electron microscopy (TEM), energy dispersive X-ray spectroscopy (EDS) lines and X-ray photoelectron (XPS) analysis [24,25]. Subsequently, we compare the I–V curves obtained using each method and conduct durability tests to assess performance. Additionally, to evaluate the device’s process accuracy and its suitability for use in device-to-device and long-term memory applications, retention tests were performed [26]. To determine the synaptic characteristics, pulse inputs were applied to extract conductance values, thus enabling the implementation of the modified National Institute of Standards and Technology (MNIST) pattern recognition using machine learning. Furthermore, spike-timing-dependent plasticity (STDP) simulations were conducted based on the Hebbian rule to explore the potential utility of these devices as nonvolatile memories [27,28].

## 2. Experimental Section

To fabricate the ITO/ZrO_x_/TaN RS device, the following process was executed: first, a commercially available SiO_2_/Si substrate was cleaned using acetone and isopropyl alcohol. On the cleaned substrate, the bottom electrode (TaN) was deposited (thickness = 100 nm) using direct current (DC) reactive sputtering using a Ta target (99.99% purity). The reactive gas was a mixture of Ar (19 sccm) and N_2_ (1 sccm) at a pressure pf 5 mTorr and a power of 350 W. On the TaN electrode, a ZrO_2_ thin film (thickness = 5 nm) was deposited by a DC reactive sputtering process using a Zr target (99.99% purity) in a gas mixture of Ar (20 sccm) and O_2_ (5 sccm). The power and the pressure of the main chamber were 200 W and 3 mTorr, respectively. On the insulating layer, a square shape (100 × 100 μm^2^) was patterned using lithography. Finally, a square-shaped top electrode (ITO, thickness = 100 nm) was obtained by deposition on an ITO target (99.99% purity) using radiofrequency (RF) sputtering and a lift-off process in acetone. Ar gas (8 sccm) was used with the main chamber pressure and the RF power of 3 mTorr and 80 W. The fabricated device’s structural and chemical compositions were determined using cross-sectional TEM images and XPS analyses in depth mode. The electrical characteristics of the ITO/ZrO_2_/TaN device were investigated using a Keithley 4200-SCS semiconductor parameter analyzer (Keithley Instruments, Cleveland, OH, USA) and a 4225-PMU pulse measuring unit (Keithley Instruments, Cleveland, OH, USA). The top ITO electrode was biased, and the bottom electrode TaN remained grounded.

## 3. Results and Discussion

**Figure 1:**
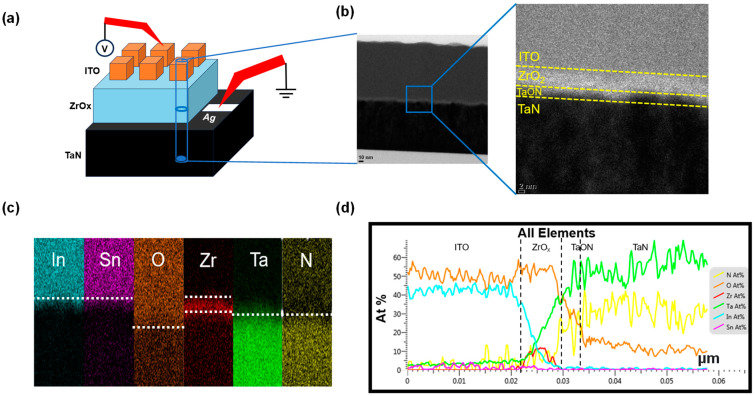
(**a**) Schematic illustration of the device structure. (**b**) Typical cross-sectional transmission electron microscopy image of the ITO/ZrO_x_/TaN structure. (**c**) Energy dispersive X-ray spectroscopy (EDS) outcomes; colors represent In, Sn, O, Zr, Ta, and N. (**d**) EDS atomic percentage composition profiles.

Figure 1a shows a schematic of the ITO/ZrO_x_/TaN structure and Figure 1b presents a cross-sectional TEM image of this structure. In the TEM image, we can observe a ZrO_x_ layer (thickness = 5 nm). A closer examination reveals that a TaON interface layer (thickness = 3 nm) between ZrO_x_ and TaN is present. This TaON interface layer is likely to be formed during the fabrication process, particularly owing to the migration of oxygen during the deposition of the ZrO_x_ layer achieved by a reactive sputter process using O_2_ and Ar gas. During the deposition of ZrO_x_, the target experiences a negative bias, while the substrate is subjected to a positive bias due to DC sputtering. As a result, Ar^+^ ions move toward the target side, while O^2−^ ions move toward the substrate side. The oxygen plasma collides with the TaN surface, thus infiltrating into the TaN layer and forming an oxygen-rich TaON interfacial layer [29]. Figure 1c depicts an EDS color map with color dots indicating the presence of the detected elements. The distinct colors help differentiate the top electrode (In, Sn, O), insulator (Zr, O), and bottom electrode (Ta, N) regions. The presence of oxygen extends up to the bottom electrode (Ta, N) part, thus indicating the existence of the TaON layer. This information is precisely corroborated by the EDS line profile in Figure 1d.

**Figure 2:**
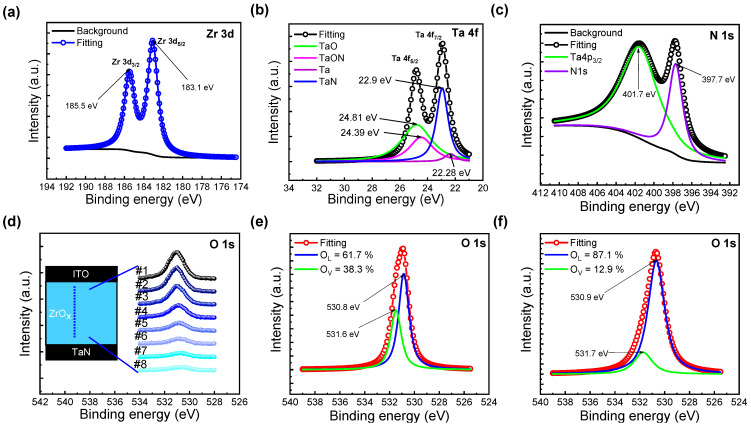
X-ray photoelectron spectra of (**a**) Zr 3d, (**b**) Ta 4f, (**c**) N 1s. (**d**) O 1s spectra of the ZrO_x_ layer at different etch times. (**e**) O 1s spectra obtained from ZrO_x_ thin films #1 and (**f**) #8.

Figure 2 presents spectra obtained based on XPS analyses. The core-level spectra for Zr 3d and Ta 4f exhibit double spin–orbit splitting; specifically, Zr 3d_3/2_, Zr 3d_5/2_, Ta 4f_5/2_, and Ta 4f_7/2_. The background has been extracted using a modified Shirley method [30]. In Figure 2a, the spin–orbit splitting of Zr 3d is equal to 2.4 eV, and the peak differences in binding energy confirm this [31]. Similarly, in Figure 2b, the response has been fitted to detectable compounds, while the spin–orbit splitting of Ta 4f is 1.8 eV [32]. The binding energy peak at 24.39 eV corresponds to TaON, thus verifying the presence of the TaON interfacial layer in the XPS analysis [33]. In Figure 2c, the N 1s peak binding energy value of 397.7 eV implies Ta-N bonds [34]. Additional explanations for the remaining figures will be provided in subsequent sections.

**Figure 3:**
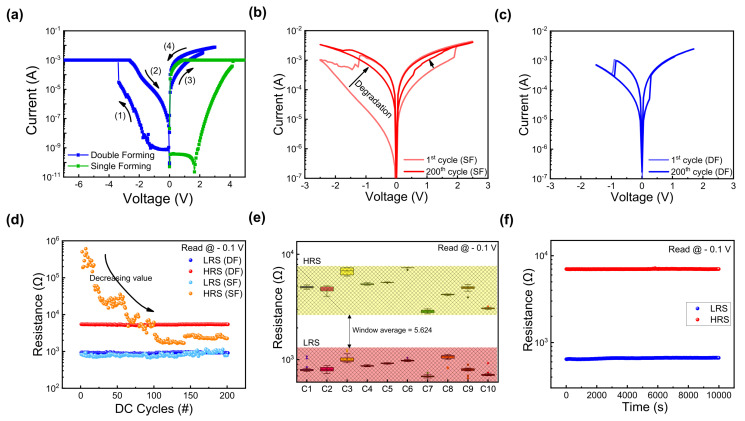
(**a**) Double-forming (DF) process of ITO/ZrO_x_/TaN. (**b**) I–V curves of 1st and 200th cycles after single forming. (**c**) I–V curves of 1st and 200th cycles after double forming. (**d**) Endurance characteristics of ITO/ZrO_x_/TaN device under different forming processes for 200 direct current (DC) cycles. (**e**) Cell-to-cell uniformity of the DF device of 10 randomly selected cells. (**f**) Retention characteristics of the DF device.

Figure 3a demonstrates the two different forming processes of fabricated ITO/ZrO_x_/TaN devices. In the case of SF, forming was performed in the voltage range of 0–5 V with a compliance current (CC) of 1 mA. Conversely, for DF, a two-step forming process was employed. In the first step, forming was performed when a bias of −7 V was applied with a CC of 1 mA. However, the current level decreases at the (2) state, thus indicating incomplete forming. In the second step, by applying a voltage bias of 3 V, the formation of a complete filament is presented. After the complete filament formation, the set and reset processes are induced by applying sequentially different polarity voltages. In Figure 3b, the device switches its resistance state in the SF case when the bias voltages of −2.5 V and 2.5 V are applied. As depicted, the variation during repeated cycles is significant, thus causing a gradual reduction in memory margin from the 1st to the 200th cycle. Conversely, in the case of DF, as illustrated in Figure 3c, the I–V curves for the 1st cycle and the 200th cycle match closely. Additionally, the operating voltage needed to induce resistive switching decreases to −1.5 V and 1.7 V, thus resulting in lower power consumption. Cycle-to-cycle endurance performance is illustrated in Figure 3d, where SF experiences a decrease in the HRS value over 200 cycles (read at −0.1 V), showing a large variance. In contrast, DF demonstrates stable switching over 200 cycles, thus maintaining a consistent resistance value. Furthermore, Figure 3e presents a box plot graph showing a uniform cell-to-cell performance in DF cells, presenting 20 DC cycles executed at 10 randomly selected ITO/ZrO_x_/TaN cells. The average ON/OFF ratio (R_HRS_/R_LRS_) of 5.624 indicates consistent performance. In Figure 3f, a retention test was conducted on the DF device to determine its lifetime. Findings demonstrate stable nonvolatile memory characteristics according to which the device maintains its resistance state for 10^4^ s at a read voltage of −0.1 V.

**Figure 4:**
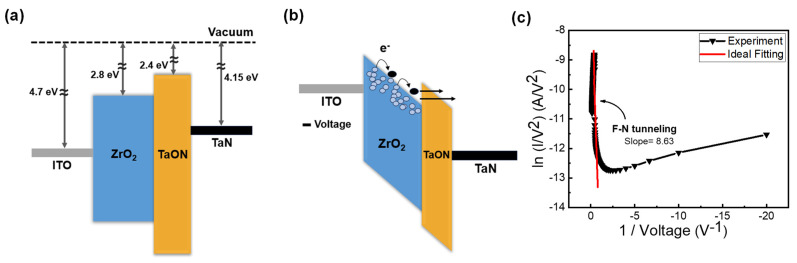
Schematic of the conduction mechanism of the DF process of the ITO/ZrO_x_/TaN resistive random access memory. (**a**) Initial state. (**b**) First forming under negative bias application. (**c**) F-N tunneling curve fitting.

In Figure 4a, the initial states of each layer and their alignment are depicted [35,36,37,38]. In the first step of DF, as illustrated in Figure 3a, when a negative voltage is applied to the top electrode, the ITO band state rises, thus creating a gradient. As this is a filamentary-type device, it allows electrons to hop through the ZrO_x_ layer following the oxygen vacancies’ defect gradient, and to tunnel through the TaON barrier, thus leading to an increase in current, as shown in Figure 4b [39]. However, when the applied bias sweeps back to 0 V, the ITO band descends again and the TaON band flattens, thickening the barrier. Consequently, electrons cannot tunnel through, thus resulting in an incomplete filament formation and failure to form in a single step. This corresponds to the part of the I–V curve labeled as state (2) in Figure 2a during which the current decreases. To verify the FN tunneling phenomenon, the I–V curve is typically plotted as ln(I/V^2^) versus 1/V, as shown in Figure 4c [40]. Conversely, when a positive voltage is applied to the top electrode, the energy band diagram from TaN to ITO forms a stepped gradient, thus allowing electrons to be hopped in a single step. This explains why positive forming is a single-step process. 

**Figure 5:**
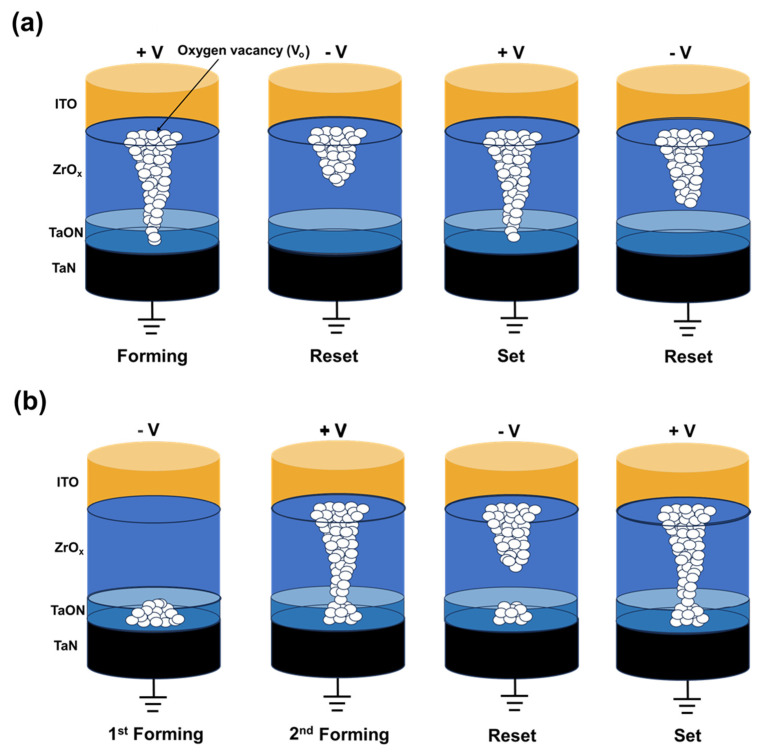
Schematic of the filament formation and rupture. (**a**) SF process. (**b**) DF process.

The mechanism of the two different forming processes is explained using a filament schematic in Figure 5. In contrast to DF, SF results in the formation of a complete filament in an SF step. To understand the reason for this, XPS etch times for O1s, detected along with Zr in Figure 2d, were divided into 8 intervals, with etch time #1 near the top electrode interface and etch time #8 near the bottom electrode interface; respective fitted outcomes are shown in Figure 2e,f. In Figure 2e, the area ratios of oxygen—in the form of O^2−^ ions in the oxygen lattice (OL) and as defects, such as V_o_^2+^ (oxygen vacancies)—were found to be 67.1% and 38.3%, respectively. In contrast, in Figure 2f, the respective ratios of oxygen-rich ZrO_x_ and oxygen-deficient interfacial TaON layers were 87.1% and 12.9% [23].

(1) $$O\begin{array}{c} \overset{Reset}{\leftarrow }\\ \underset{Set}{\to } \end{array}V_{O}^{2+}+O^{2-}$$

Based on the electrical and chemical characteristics of ITO/ZrO_x_/TaN, the conduction mechanism is proposed based on the migration of oxygen ions, as shown in Figure 5. In the case of the SF process (Figure 5a), when a positive bias is applied to the top electrode ITO, oxygen ions migrate toward the oxygen-reserving ITO electrode, thus leaving oxygen vacancies. Under continuous voltage stress, these vacancies accumulate and form a conducting filament, thus connecting the ITO and TaN electrodes. A large current flows through the conductive path, thus resulting in a resistance state change, from the initial resistance state (IRS) to an LRS. When an opposite bias is applied to the top electrode, the oxygen ions that migrate to the ITO electrode are repelled owing to the negative bias; correspondingly, they return to their original location. Through the migration of oxygen ions, recombination of oxygen ions and vacancies occurs, thus rupturing the conductive filament. However, during the repeatable switching, as shown in Figure 3d, degradation of device performance occurs in the SF mechanism. This may be owing to the inherent randomness of the conducting filament that results in different widths and the robustness of the conductive filament. Furthermore, the decreasing HRS of the SF device may be interpreted as the hard breakdown process occurring in the ZrO_x_ film owing to the major voltage drop in this area [41].

Conversely, the mechanism of DF incorporates a two-step forming process to induce resistive switching phenomena in ITO/ZrO_x_/TaN devices. When a negative voltage is applied to the top electrode, oxygen ions migrate toward the TaN electrode, thus resulting in the breakdown of the TaON layer. However, during the process, the conductive filament is not completely formed (as in Figure 5b) owing to the insufficient CC [42]. When a positive bias is applied to the top electrode, oxygen ions migrate toward the ITO electrode, thus leaving oxygen vacancies and creating a conductive filament. This leads to an hourglass-shaped conductive path, with its weakest point located at the interface of ZrO_x_ and TaON. Owing to this weak interface conductive filament, the RS phenomenon occurs in this area. This leads to uniform rupture and generation of conducting filaments, thus improving device-switching performance [43].

**Figure 6:**
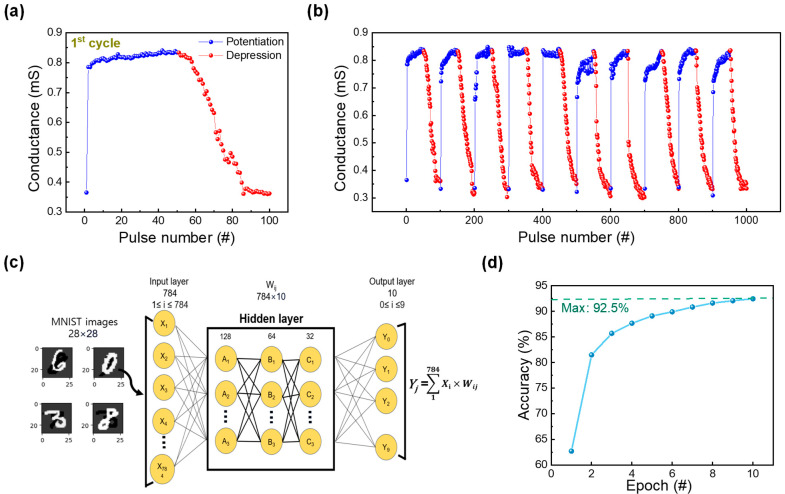
(**a**) Potentiation and depression curves of the ITO/ZrO_x_/TaN device. (**b**) Ten consecutive potentiation and depression cycles of ITO/ZrO_x_/TaN device. (**c**) Deep neural network simulation framework for modified National Institute of Standards and Technology (MNIST) pattern recognition. (**d**) Plot of pattern recognition accuracy of a synaptic device quantified at 10 epochs.

The uniform operation (occurring at low voltages) achieved based on the DF process is sufficient to utilize this RRAM device for neuromorphic applications. To confirm the synaptic characteristics, identical pulse tests were conducted (presented in Figure 6a). A voltage pulse (with an amplitude of 2.2 V and a width of 20 μs) was applied followed by a read voltage at 0.1 V to observe the change in conductance values; the process was repeated 50 times. Similarly, 50 consequent depression pulses were applied to decrease the conductance value of the device. The depression consisted of a reset pulse (amplitude and width of −2 V and 50 μs, respectively) also followed by a read pulse to observe a decrease in conductance value. Furthermore, the reproducibility of this behavior was tested by rehearsing this pulse application 10 times, as illustrated in Figure 6b [44]. Subsequently, the potentiation and depression curves of the ITO/ZrO_x_/TaN device were used (to extract weights) in a neural network for a pattern recognition system. The conductance values in Figure 6a were applied to $\frac{G-G_{min}}{G_{max}-G_{min}}$, where *G_max_* and *G_min_* represent the maximum and minimum conductance values, respectively. Using this method, the conductance of potentiation and depression was converted into a 28 × 28-pixel handwritten number image based on MNIST, where the rise and fall of changes in conductance refer to the white and black pixels of the handwritten image. To conduct pattern recognition tests, a deep neural network that consisted of three layers of input, hidden, and output layers was used. Among the layers, the hidden layer was subdivided into an additional three layers, each having nodes of 128, 64, and 32, as depicted in Figure 6c. After training the machine learning model for 10 epochs and testing it with 10,000 images, it achieved an accuracy of 92.5%, as shown in Figure 6d.

**Figure 7:**
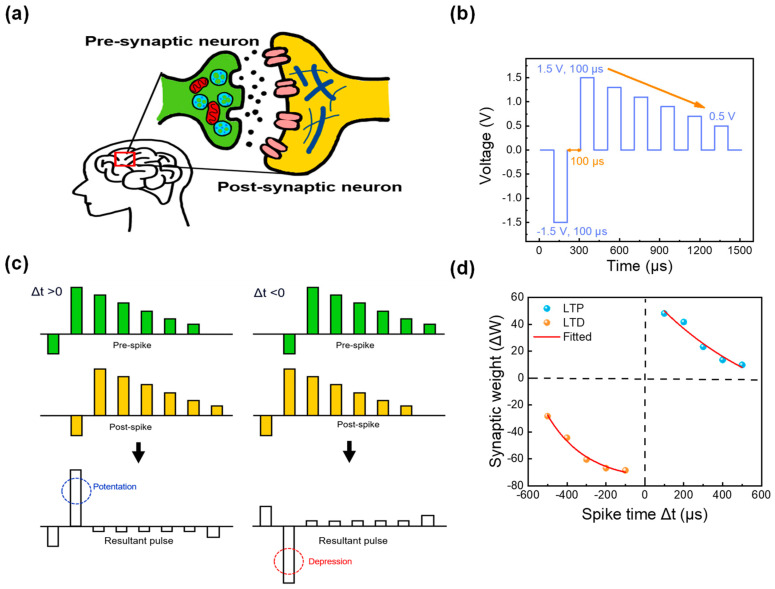
(**a**) Schematic of human synaptic neural structure, (**b**) Pulse schematic, (**c**) Pulse authorization for spike-timing-dependent plasticity (STDP) measurements at Δt = 100 μs, (**d**) STDP measurement outcomes.

Finally, to implement and emulate synaptic responses, the Hebbian learning rule was applied to a neural network. One of the key features of the Hebbian learning rule is STDP. The STDP mechanism plays a crucial role in memory and learning in neural networks by regulating the connection strength of neurons. In the STDP mechanism, when presynaptic neurons receive stimuli, they transmit electrical signals known as spikes. When these spikes reach the synapse, neurotransmitters are released, thus activating the synapse. The postsynaptic neuronal receptors then detect this activity, and a spike is generated in the postsynaptic neuron after the synapse (that is, signal transmission occurs from presynaptic to postsynaptic neurons). This process is depicted in a simplified diagram in Figure 7a [45]. Owing to the simple two-terminal feature of the RRAM device, the biological synapse can be easily mimicked in the ITO/ZrO_x_/TaN device, where all pre- and postsynaptic parts are emulated by ITO and TaN layers as the top and bottom electrodes. To implement the fired spike and the time intervals of firing between pre- and postsynaptic regions, a pulse train shown in Figure 7b was applied to the top and the bottom electrodes at different time points. The pulse consisted of a pulse interval and width equal to 100 μs at the voltages of −1.5, 1.5, 1.3, 1.1, 0.9, 0.7, and 0.5 V [46]. At different time circumstances (spike time), the same pulses were applied to the top and bottom electrodes as those in Figure 7c. When the presynaptic spike amplitude exceeds that of the postsynaptic spike (Δt > 0), potentiation occurs, and the synaptic connections are strengthened. Conversely, when the postsynaptic spike exceeds the presynaptic spike amplitude (Δt < 0), depression occurs, and synaptic connections are inhibited. The term “spike time” refers to the difference in spike firing times of the pre-and postsynaptic regions (Δt = t_pre_ − t_post_). The resultant pulse, obtained by subtracting the postsynaptic from the presynaptic spike, was measured. When the interval time is shorter (that is, when the gap between spikes is smaller), it is perceived that there is a higher correlation between neurons, thus leading to significant changes in synaptic connections and transitioning into longer-term memory mechanisms. The result of the STDP function is illustrated in Figure 7d. 

The term synaptic weight (Δ*W*) refers to the following equation:

(2) $$\Delta W=\frac{G_{f}-G_{i}}{G_{i}}\times 100\;\left(\%\right)$$

where the terms *G_f_* and *G_i_* represent the values of conductance before and after pulse application. The fitted curve for the STDP function was obtained using the following equation:

(3) $$y=A_{1}\times \exp\left(\frac{x}{t_{1}}\right)+y_{0}$$

where the terms *A*_1_, *y*_0_, and *t*_1_ are equal to 4.064, −76.569, and −2.007 × 10^−4^ for LTD, and 105.745, −40.356, and −6.382 × 10^−4^ for LTP, respectively. As illustrated by the results, gradual synaptic weight changes as a function of spike time can be observed following the STDP function.

## 4. Conclusions

In this study, we confirmed that the ITO/ZrO_x_/TaN RRAM device can form filaments using two different methods based on TEM and EDS line energy band diagrams and XPS analysis. Among these methods, the DF device exhibits lower current variation and low-power operation, thus making it more suitable for measuring synaptic characteristics in neuromorphic systems. First, we implemented pattern recognition for the MNIST dataset using identical pulses and machine learning. Second, we explored the applicability of this semiconductor as a neuromorphic chip mimicking the human brain. Based on STDP measurements, we observed varying degrees of long-term memory based on spike-time intervals, thus indicating its potential suitability as a long-term memory element in neuromorphic systems.

## Data Availability

Not applicable.
